# eTumorMetastasis: A Network-based Algorithm Predicts Clinical Outcomes Using Whole-exome Sequencing Data of Cancer Patients

**DOI:** 10.1016/j.gpb.2020.06.009

**Published:** 2021-02-11

**Authors:** Jean-Sébastien Milanese, Chabane Tibiche, Naif Zaman, Jinfeng Zou, Pengyong Han, Zhigang Meng, Andre Nantel, Arnaud Droit, Edwin Wang

**Affiliations:** 1 Human Health Therapeutics, National Research Council Canada, Montreal H4P 2R2, Canada; 2 Genomics Center, Centre Hospitalier Universitaire de Québec - Université Laval Research Center, Quebec G1V 4G2, Canada; 3 Department of Biochemistry & Molecular Biology, Medical Genetics, and Oncology, University of Calgary, Calgary T2N 4N1, Canada; 4 Alberta Children’s Hospital Research Institute and Arnie Charbonneau Cancer Research Institute, University of Calgary, Calgary T2N 4N1, Canada; 5 Institute of Biotechnology, Chinese Academy of Agricultural Sciences, Beijing 100086, China; 6 Department of Medicine, McGill University, Montreal H3G 2M1, Canada

**Keywords:** Breast cancer, Sequencing data, Predictive model, Systems biology, Machine learning

## Abstract

Continual reduction in sequencing cost is expanding the accessibility of genome **sequencing data** for routine clinical applications. However, the lack of methods to construct **machine learning**-based **predictive models** using these datasets has become a crucial bottleneck for the application of sequencing technology in clinics. Here, we develop a new algorithm, eTumorMetastasis, which transforms tumor functional mutations into network-based profiles and identifies network operational gene (NOG) signatures. NOG signatures model the tipping point at which a tumor cell shifts from a state that doesn’t favor recurrence to one that does. We show that NOG signatures derived from genomic mutations of tumor founding clones (*i.e.*, the ‘most recent common ancestor’ of the cells within a tumor) significantly distinguish the recurred and non-recurred breast tumors as well as outperform the most popular genomic test (*i.e.*, Oncotype DX). These results imply that mutations of the tumor founding clones are associated with tumor recurrence and can be used to predict clinical outcomes. As such, predictive tools could be used in clinics to guide treatment routes. Finally, the concepts underlying the eTumorMetastasis pave the way for the application of genome sequencing in predictions for other complex genetic diseases. eTumorMetastasis pseudocode and related data used in this study are available at https://github.com/WangEdwinLab/eTumorMetastasis.

## Introduction

As genome sequencing is becoming cheaper and more convenient, it is now more accessible for routine clinical usage and its demand is rising. It has been expected that massive genome sequencing data combined with phenotypic and complex disease data will allow us to decode the underlying molecular mechanisms of diseases, and to predict clinical outcomes. Moreover, to fulfill the promises of precision medicine, it is necessary to construct clinically useful predictive models using DNA sequencing data. However, using these datasets to construct such predictive models has become a crucial bottleneck in genomic biomarker development. Thus far, none of the existing machine learning algorithms is suitable to construct predictive models for diseases from genome sequencing data alone. For example, in a recent Dialogue for Reverse Engineering Assessment and Methods (DREAM) effort, scientists have tested more than 50 existing prediction algorithms and shown that none of them is able to construct cancer drug-response predictive models using solely genome sequencing data [1].

The huge challenge we are facing when constructing predictive models using genome sequencing data is that complex diseases are often modulated by multiple distinct genetic pathways. For a given phenotype (i.e., a complex disease), there are many ways to produce the phenotype, each of which is formed by the combined effects of multiple genes whose functions can be modulated through either genetic or epigenetic changes. Thus, different individuals who have the same phenotype/disease may have different causal genes and thus, may express different optimal drug targets. As an added level of complexity, tumors often exhibit extensive mutational heterogeneity, with genes’ mutation status varying widely across individual cancer cells. In other words, mutated genes are rarely shared between any two individual tumors of even a single cancer type and each patient has an individually unique genomic profile [2], [3], [4], [5], [6], [7], [8], [9], [10], [11], [12]. This feature of tumor mutations makes it extremely challenging to apply machine learning approaches for accurately predicting clinical outcomes based only on their genomic DNA.

Traditionally, clinical factors and histology were used to assess patient’s relapse risk in many cancer types. Due to many variables, such as tumor heterogeneity, these factors have poor predictive power, which results in many patients being misclassified and ultimately leads to overtreatment (or recurrence). However, recent efforts have been made to improve decision making about treatment options. Gene expression profiling assays, such as Oncotype DX and MammaPrint, are now being used in a variety of countries and can help estimate benefit from chemotherapy as well as the risk of recurrence [13], [14]. As a result, chemotherapy use for early breast cancer has now dropped by 26.6% [15]. However, these findings also suggest that 46% of women still do not benefit from chemotherapy, highlighting the importance of identifying low-risk accurately and emphasizing the utility of genomic assays.

To cope with these challenges, we develop a novel network-based method to construct predictive models using the collective impact of genomic alterations in tumors, focusing on functionally mutated genes. We model the tipping point when a system shifts abruptly from one cellular state to another. As a proof-of-concept, we develop an algorithm, eTumorMetastasis, which predicts tumor recurrence using tumor whole-exome sequencing data. We also demonstrate that mutations in the tumor founding clones (*i.e.*, the founding cancer cell transformed from a single normal cell through the acquisition of a series of mutations) can be used to predict tumor recurrence in breast cancer. We show that eTumorMetastasis outperforms Oncotype DX, the most popular genomic test for breast cancer [16], [17]. Finally, by using genome/whole-exome sequencing data, we envision that this method could be widely applicable to constructing predictive models for other cancer types and other complex genetic diseases (or phenotypes) to improve personalized medicine and current cancer treatment protocols.

## Method

### Data for tumors and paired normal samples

Whole-exome sequencing data of estrogen receptor-positive (ER^+^) breast tumors and their paired normal samples were obtained from The Cancer Genome Atlas (TCGA). Patients with clear clinical information on recurrence and clinical follow-up were selected for the analysis. Based on these criteria, we retained 400 ER^+^ breast tumor samples (released by TCGA in 2012; hereafter referred to as “TCGA-Nature set”). From TCGA-Nature set, we randomly selected 200 samples to build our training set. The 200 remaining samples from TCGA-Nature set were used as a validation set. Further, we obtained data from 295 ER^+^ breast tumor samples in the cBioPortal (https://cbioportal.org) which was released in 2017 by The Clinical Proteomic Tumor Analysis Consortium (TCGA-CPTAC). In total, data from 695 ER^+^ breast tumor samples (*i.e.*, 200 from TCGA-Nature as the training set, 200 from TCGA-Nature as the validation set 1, and 295 from TCGA-CPTAC as validation set 2) were used in this study (Tables S1–S3). Gene expression profiles and Affymetrix SNP 6.0 array data from these samples were also downloaded from TCGA and cBioPortal. The samples/data were processed following examination of the tumor purity and variant calling (File S1). The sample numbers remaining after each processing step in the training and validation sets are listed in Table S2.

### Identification of mutations in germline and founding clones

Based on tumor purity, sequencing reads from each variant were adjusted accordingly, and then the variant allele frequency (VAF) was recalculated (File S1). Only mutations in 2n regions of chromosomes (*i.e.*, excluding the amplified and deleted regions) were considered. Germline mutations included: 1) a homozygous mutation with VAF ≥ 90% in both normal and tumor samples; and 2) a heterozygous mutation with 55% ≥ VAF ≥ 45% in normal samples. Founding clone’s somatic mutations included: 1) a homozygous mutation with VAF ≥ 90% in tumor but not in its paired normal sample; and 2) a heterozygous mutation with 55% ≥ VAF ≥ 45% in the tumor sample but not in its paired normal sample. This process was applied to each sample independently. Finally, we obtained a total of 695 founding clones (*i.e.*, one founding clone per sample). To further validate our selected VAF cutoffs, we changed values for homozygous mutations (*i.e.*, VAF ≥ 95%) and heterozygous mutations (*i.e.*, 60% ≥ VAF ≥ 40%). Using these cutoffs, we re-ran eTumorMetastasis and obtained similar results.

### Determination of functionally mutated genes

To determine whether a genetic variant is functionally mutated, we applied the following tools: CRAVAT (*i.e.*, a functional mutation is defined as a score ≥ 0.5), MutationTaster2 (*i.e.*, a functional mutation is defined as having a disease impact), and Combined Annotation Dependent Depletion (CADD; *i.e.*, C-score 10 is set as a cutoff and a missense variant is predicted as damaging or deleterious by PolyPhen-2 or SIFT) [18], [19], [20], [21], [22]. For a given sample, we merged all functional mutations predicted by the three tools for further analysis. In this study, all the mutated genes mentioned are referred to as ‘functionally mutated genes’. In each founding clone, the average number of somatic mutations in coding regions and the functionally mutated genes defined by each tool are listed in our GitHub directory (https://github.com/WangEdwinLab/eTumorMetastasis). Table S4 contains the complete matrix of all functional mutated genes in each sample.

### Construction of an ER^+^ breast cancer-specific recurrence network

To construct an ER^+^ breast cancer-specific recurrence network, we modified the procedure for constructing ER^+^ breast cancer-specific survival and proliferation networks [11]. Briefly, we extracted a subnetwork by mapping the ER^+^ breast cancer-specific recurrence-associated genes onto the literature-curated human signaling network. To do so, we first identified recurrence-associated genes using ER^+^ cancer cell lines and tumor samples. We obtained gene expression data of 22 ER^+^ cancer cell lines from the Cancer Cell Line Encyclopedia (CCLE; https://www.broadinstitute.org/ccle). Gene expression data normalization was conducted using median centering and z-score normalization method described previously [2]. Using the ratio of two genes’ expression values (*i.e.*, *CDH1*/*VIM* for determining epithelial–mesenchymal transition), we classified these cell lines into epithelial (*n* = 13, *CDH1*/*VIM* > 1.2) and mesenchymal (*n* = 9, *CDH1*/*VIM* ≤ 1.2) lines. Modulated genes (called Set 1) were identified by conducting *t*-test comparison (*P* < 0.05) of the two groups of cell lines with 10 re-samplings (*i.e.*, for each re-sampling we randomly selected 60% of the original samples). We also obtained gene expression data of 1197 ER^+^ tumor samples from METABRIC set (https://www.ebi.ac.uk/ega/studies/EGAS00000000083) which had information about cancer recurrence and clinical follow-up. Using this set, we used a *t*-test (*P* < 0.05) to identify modulated genes between the recurred and non-recurred samples. Next, we performed Kaplan-Meier survival tests on these modulated genes to identify survival-associated genes (called Set 2) using 10 re-samplings. We further identified potential cancer regulator genes by analyzing copy number data (SNP 6.0) of the ER^+^ tumor samples from TCGA. SNP 6.0 data were processed using GISTIC2 to obtain GISTIC scores for each gene. For a given gene in a sample, if its GISTIC score was greater than 0.3 and its expression value was ranked among the top 50% of the genome, we defined this gene as a cancer regulator (for details, see Zaman et al. [10]). The cancer regulator genes of all TCGA’s ER^+^ tumor samples were defined as Set 3. Genes from Sets 1, 2, and 3 were mapped onto the signaling network. In other words, we merged the manually curated human signaling network and the protein–protein interaction network. Network genes common in all three sets and their respective links were retained to obtain an ER^+^ breast cancer-specific recurrence network that contains 6148 genes and 62,004 interactions (see our GitHub directory for a complete list of the genes used and the network, https://github.com/WangEdwinLab/eTumorMetastasis).

### Generation of network profiles using a network propagation approach

To generate a network profile for a sample, we projected its mutated genes as seeds onto the ER^+^ breast cancer-specific recurrence network and then applied the network propagation algorithm to obtain heating scores of the genes within the network [23]. This process is named network profiling, and the set of resulting heating scores for all the network genes for a tumor sample is called a netProfile. Then, we applied a scaling factor of 100,000 to the heating scores and then conducted data transformation using median centering and z-score within sample approach [2]. To examine the potential effects of the scaling factor, we conducted a sensitivity analysis by re-running the eTumorMetastasis using the scaling factor of 10,000. We found that the prediction accuracies of the gene signatures were similar using both scaling factors. Table S5 contains all the network propagation scores for each gene and each sample. For future reference, a combination of netProfiles is referred to as a netMatrix.

### Pseudocode for eTumorMetastasis

**Figure 1:**
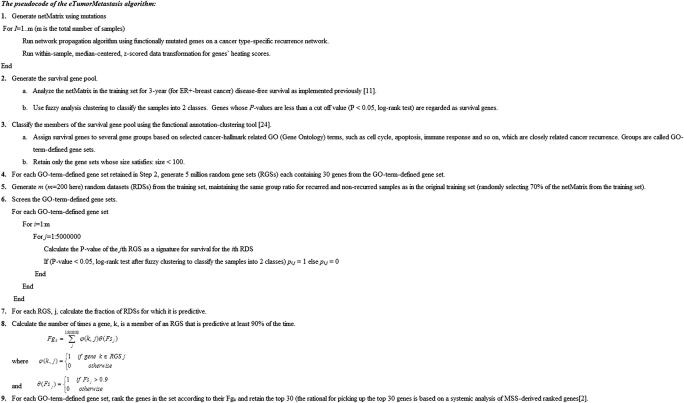
**The pseudocode for the eTumorMetastasis algorithm** For Gene Ontology annotation, we used the DAVID [24]. GO, Gene Ontology; RGS, random gene set; RDS, random dataset; MSS, Multiple Survival Screening; DAVID, Database for Annotation, Visualization and Integrated Discovery.

Multiple Survival Screening (MSS) was originally designed for identifying gene signatures using tumor gene expression profiles [2]. Based on clinical follow-up information, we used the netMatrix of our training set to run MSS. However, the resulting network operational gene (NOG) signatures failed to predict tumor recurrence in the validation sets, suggesting that they were not sufficiently robust. In contrast, by using the same MSS procedure to the tumor gene expression profiles of the same training set, we obtained several gene signatures that could successfully predict tumor recurrence in the validation sets. Together, these results suggest that gene heating scores (propagation scores) in the netMatrix and gene expression profiles are somewhat different. The gene heating scores in the netMatrix, by themselves, are perhaps too weak or too noisy to successfully identify NOG signatures using traditional machine learning algorithms. The pseudocode of the eTumorMetastasis algorithm is shown in Figure 1.

#### Identification of the NOG signatures

NOG signatures were obtained using our previously developed method MSS [2]. The netMatrix (described above) is a simple matrix, where rows represent genes, columns represent samples, and values represent heating scores. The samples were separated into two groups (recurred and non-recurred). To obtain NOG signatures, we applied MSS to this netMatrix. Briefly, we used fuzzy clustering to classify the netMatrix into two classes and then conducted a log-rank test to identify modulated genes (*P* < 0.05) by using network propagation scores. From these modulated genes, we collected hallmark Gene Ontology (GO) term-defined genes which resulted in 100–200 genes for every cancer hallmark (apoptosis, cell cycle, cell adhesion, cytoskeleton, immune response, and cell proliferation). From the training set, we generated 200 random netMatrix sets (random sample sets) and also generated 5 × 10^6^ random gene sets (30 genes per set) from the GO term-defined gene list. Then, we ran a fuzzy clustering analysis (*k* = 2) for each random netMatrix sets (200) using each gene set (5 × 10^6^) to distinguish low- and high-risk groups (log-rank test, *P* < 0.05). For every cancer hallmark, we retained all random gene sets that were able to distinguish low- and high-risk groups (log-rank test, *P* < 0.05) in more than 80% of the 200 random netMatrix sets (*P* < 0.05), ideally resulting in 1000–5000 total gene sets. If the number of gene sets was lower than 1000, the signature obtained from the gene frequency was not robust enough and was discarded. If the number of gene sets was greater than 5000, we set a more stringent *P* value cutoff. Finally, we calculated the frequency of all genes in gene sets retained and the top 30 genes were used as a NOG signature. More details are described in our previous publication on MSS [2].

### Construction of combinatory NOG signature sets

**Figure 2:**
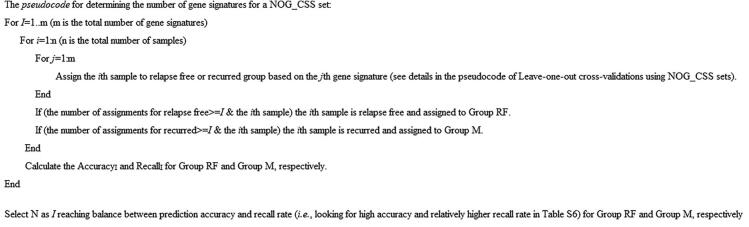
**The pseudocode for determining the number of gene signatures for a NOG_CSS** NOG_CSS, combinatory network operational gene signature set.

This procedure was modified from our previous study [3]. To determine the number of the NOG signatures for a combinatory NOG signature set (NOG_CSS), we systematically tested combinatory predictions using *N* (*N* = 1, 2, 3, …) of gene signatures. *N* is defined as the number of gene signatures in a NOG_CSS to reach higher prediction accuracy and relatively higher recall rate for the samples (Table S6). We selected *N* when the predictive accuracy could not be significantly improved and when the recall rate could be significantly dropped by adding one or more additional gene signatures to the NOG_CSS. The NOG_CSSs were identified by combining the training set and the cutoff set (60 randomly selected samples). Combining sets with unique samples help to avoid bias toward the training set when constructing NOG_CSSs. The pseudocode for constructing NOG_CSSs is shown in Figure 2.

### Leave-one-out cross-validations using NOG_CSSs

**Figure 3:**
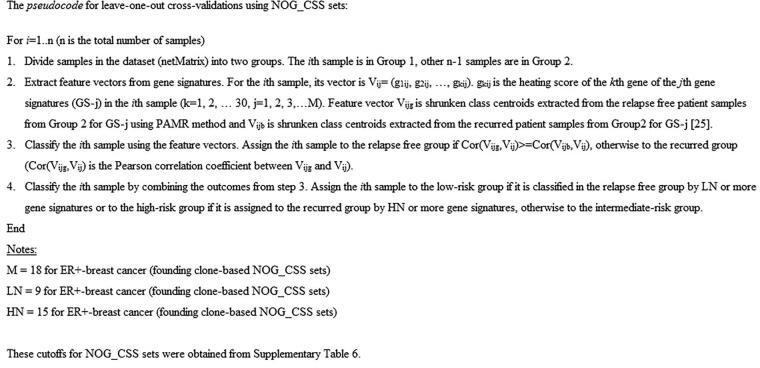
**The pseudocode for leave-one-out cross-validations using NOG_CSSs** *M* = 18 for ER^+^ breast cancer (founding clone-based NOG_CSSs); LN = 9 for ER^+^ breast cancer (founding clone-based NOG_CSSs); HN = 15 for ER^+^ breast cancer (founding clone-based NOG_CSSs). These cutoffs for NOG_CSSs are obtained from Table S6. PAM50, Prediction Analysis of Microarray 50; LN, low-risk signature cutoff number; HN, high-risk signature cutoff number.

This procedure was also modified from our previous study [3]. Each sample was cross validated by each gene signature of a NOG_CSS using the algorithm described previously [2]. We used shrunken-class centroids combined with Prediction Analysis of Microarray 50 (PAM50) method to perform leave-one-out cross-validations [25], [26]. The pseudocode for leave-one-out cross-validations using NOG_CSSs is shown in Figure 3.

### Performance comparison between Oncotype DX and eTumorMetastasis

The Oncotype DX breast cancer test is the most popular genomic test for cancer prognosis. It assesses the recurrence risk and whether a patient will benefit from chemotherapy treatment. The test uses the expression values of 21 genes to calculate a recurrence score (RS) for ER^+^ breast cancer patients using a formula (File S1) [13], [16], [17]. Gene expression values can be obtained from microarray, RT-PCR, or RNA-seq [27]. Based on the RS, a patient will be assigned into low-, intermediate-, or high-risk. As a comparative analysis, we applied the Oncotype DX formula to our dataset using the normalized RNA-seq data downloaded from the GDC [fragments per kilobase of transcript per million mapped reads (FPKM)-UQ, 751 samples in total). The prediction results of all samples obtained from eTumorMetastasis and Oncotype DX are shown in Table S7. Additional comparisons with raw read counts (HTSeq) and FPKM can also be found in Tables S8 and S9.

## Results

### An overview of the eTumorMetastasis

Tumor recurrence is the leading cause of cancer mortality, and an accurate evaluation of this process could greatly aid clinicians in making treatment decisions. For example, most of the low-risk breast cancer patients (*i.e.*, patients whose tumors would not recur for 10 years after surgery alone) do not gain survival benefits from adjuvant therapy (*i.e.*, chemotherapy given after surgery to reduce the risk of cancer recurrence), but will suffer from its toxic side effects. Therefore, it is essential to identify gene markers that are able to accurately identify low-risk cancer patients who do not require adjuvant chemotherapy. The ITRANSBIG Consortium (https://www.breastinternationalgroup.org) suggests that, to be clinically practicable, low-risk patients should be associated with 10-year overall survival probabilities of at least 88% for ER^+^ tumors. Prognostic biomarkers could predict whether a patient is more likely to suffer from tumor recurrence and whether they would benefit from adjuvant chemotherapy.

**Figure 4:**
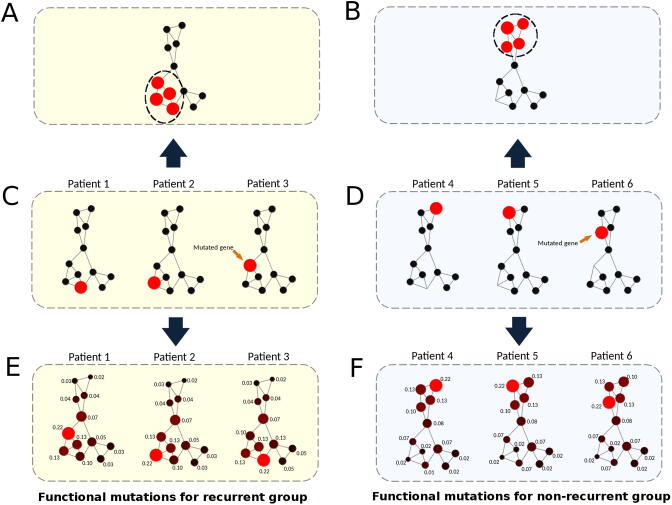
**Network propagation for recurred and non-recurred samples** **A.** Network clusters for recurred samples in the human signaling network. **B.** Network clusters for non-recurred samples in the human signaling network. **C.** Different functionally mutated genes in three recurred samples forming a network cluster in (A). **D.** Different functionally mutated genes in three non-recurred samples forming a network cluster in (B). **E.** For each recurred sample, by conducting network propagation based on its mutated genes, a network cluster emerges and is similar to the cluster in (A). **F.** For each non-recurred sample, by conducting network propagation based on its mutated genes, a network cluster emerges and is similar to the cluster in (B). The network clusters for the recurred group (E) and the non-recurred group (F) make it possible to classify recurred and non-recurred samples, respectively. Nodes and lines in the network represent genes and gene interactions, respectively. Numeral number of each network node represents the propagation score obtained from network propagation (see Method). Red nodes represent mutated genes while propagated nodes are represented in garnet. Node sizes are proportional to the values of the propagation scores.

To develop predictive models for cancer prognosis, machine learning requires the identification of features that distinguish recurred and non-recurred cancer patient groups, *i.e.*, genes which are frequently functionally inactivated/activated within the recurred group but not in the non-recurred group, and vice visa. We and others previously showed that functional cancer mutations collectively affect several network regions or subnetworks of the human signaling network (Figure 4**A and B**) [2], [11], [23]. Because the mutation profile of tumors at the gene level is sparse (Figure 4C and D), this type of data by itself is not suitable for machine learning approaches. These results suggest that cancer signaling processes triggered by the mutations of the recurred (or non-recurred) samples are convergent to several subnetworks or network regions (Figure 4E and F). Consequently, while recurred (or non-recurred) samples share certain impaired signaling processes/subnetworks, they do not necessarily share the same sets of mutated genes (*i.e.*, there are many ways to “break” a subnetwork). These network regions or clusters may represent key cancer signaling processes underlying the molecular mechanisms of the samples in either the recurred or non-recurred group. Therefore, we envisioned that if we could use the mutations of a tumor to infer which network regions or clusters are functionally impaired, then we can identify shared features within the tumor samples of either the recurred or the non-recurred group.

Computational techniques such as random walk and network propagation enable us to transform mutations of a tumor into the perturbed signaling network regions or clusters in the human signaling network. The network propagation algorithm works by projecting the mutated genes of a tumor onto a cancer type-specific recurrence network in which each mutated gene is represented as a heat source. The heat source diffuses to neighboring genes along the edges of the network in a process which is analogous to heat diffusion. After a certain time period, the diffusion stabilizes to a point where each gene in the network will have received a certain amount of ‘energy’, which is represented by a ‘heating’ score or propagation score. A heating score could be treated in a similar fashion to a transcript abundance value for that gene. The higher a heating score is, the more functionally active (or inactive) the gene is. Thus, we expected that the genes in the common network regions of either the recurred or non-recurred group will have higher but similar heating scores (Figure 4E and F). Further, we appended the netProfile of each tumor to form a netMatrix. By doing so, we transformed a sparse gene mutation dataset into a data-richer matrix containing data similar to gene expression profiles.

From a systems biology perspective, a signaling network has a critical transition threshold (*i.e.*, a tipping point) at which point the system shifts abruptly from one state to another [23]. The critical transition threshold for a recurrence network could be marked by an abrupt change between the cellular states that favor or not tumor recurrence [28]. Although cancer driver-mutated genes are highly diverse between tumors, for a recurred tumor, their collective effects on the recurrence network could converge to trigger a state’s switch (*e.g.*, leading to a phenotypic switch) from the non-recurring state to the recurrence-promoting state. We thus propose a NOG signature to quantify the two cellular states and the state’s switch. A NOG signature contains a set of genes whose propagation scores (*i.e.*, the mean of the scores) in the recurred samples and non-recurred samples represent the recurring and non-recurring cellular states, respectively. Further, these scores are not only far from the tipping point (*i.e.*, state switch which is assumed to be the middle point of the distance between the two states) between the recurred and non-recurred states, but also significantly different between the two groups.

To identify the NOG signatures, we could apply the netMatrix algorithms that have historically been used for classifications based on gene expression profiles. However, unlike gene expression profiles, gene heating scores in the netMatrix are too weak to produce high-quality NOG signatures from traditional machine learning algorithms. Therefore, we modified our previously developed MSS algorithm to successfully identify NOG signatures which could significantly distinguish recurred and non-recurred tumors [2]. Finally, for the whole-exome sequencing data of a given new tumor sample, we calculated a netProfile and correlated it with the heating score profiles of the two states of the NOG signatures to successfully predict its prognosis. For example, if the netProfile is far from the tipping point and close to the heating score profile of the recurred state for a NOG signature, then we would assign that tumor to the recurred group. The implementation of these ideas (*i.e.*, eTumorMetastasis) has been described in Method.

### Tumor founding clone mutations predict tumor recurrence

**Figure 5:**
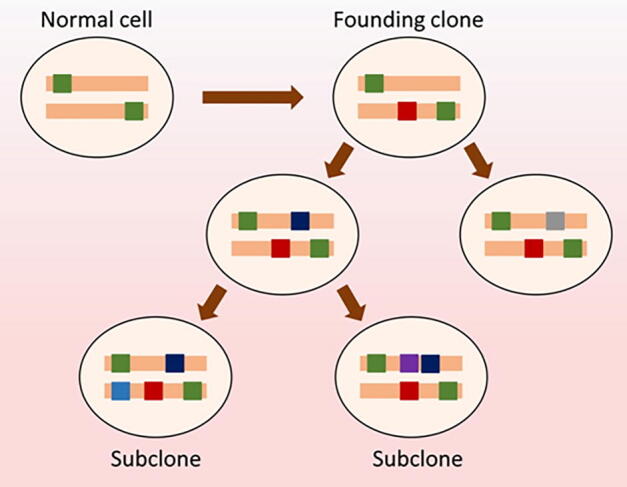
**Tumor evolution — a founding clone and its mutations** New somatic mutations (red boxes) functionally or epistatically work with germline mutations (green boxes) to form a founding clone (*i.e.*, the earliest, ancestral cancer cell). New somatic mutations (dark blue, light blue, gray, and purple boxes) occur in the founding clone to generate subclones. A tumor often contains several subclones. Of note, all the mutations from germline and somatic mutations in the founding clone are present in all the subclones and every cancer cell of the tumor. Circles represent cells while colored boxes represent mutated genes. Criteria to define founding clone mutations are explained in Method.

To test if genome/whole-exome sequencing data can be used to robustly predict cancer prognosis, we used mutations identified in the tumor founding clones. A single normal cell could acquire a set of random mutations that allows it to be transformed into the founding cancer cell (*i.e.*, the founding clone), an early evolutionary stage of a tumor. Additional accumulation of mutations from this founding clone leads to the formation of a tumor that is composed of a heterogeneous mixture of cells (Figure 5). Therefore, each tumor originates from a founding clone whose mutations are ubiquitously present in all the cells of that tumor. New mutations do not arise in isolation but rather act together in a complementary manner with the established genomic landscape. Therefore, the pre-existing mutations or genetic variants of a molecular network may have a profound effect on cellular fate and determine whether novel mutations will result in altered cell death modulation, clonal expansion, tumor recurrence, and other cancer hallmark traits. This implies that the genetic makeup of the founding clone provides an evolutionary constraint for sequential subclones and limits the genetic and clonal complexity of tumors. Tumor genome sequencing and the frequencies of the observed mutations allow us to dissect the mutations of a founding clone and its subclones and to replay the tape of the tumor’s evolutionary history, while eTumorMetastasis (see Method and File S1) allows us to decipher the tumor evolution and patient outcomes that are affected by these early mutational events. Therefore, we examined whether the sum of somatic mutations in the founding clones could predict tumor recurrence.

**Figure 6:**
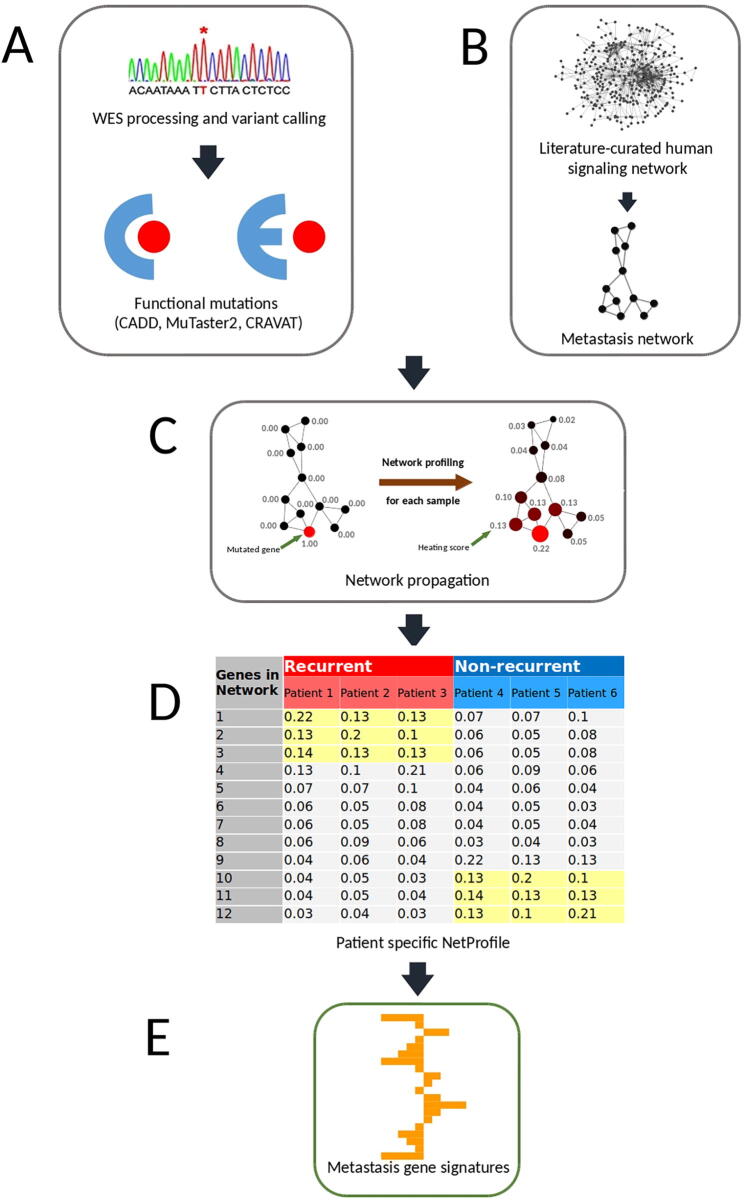
**A flowchart of eTumorMetastasis** **A.** Functional mutations are identified using whole-exome sequencing data of tumors and their paired normal samples. **B.** A cancer-specific metastasis network is constructed using the gene expression data associated with cancer recurrence and the literature-curated signaling network. **C.** Functionally mutated genes of samples are projected on a recurrence network to generate gene heating scores using a network propagation approach. In the network, nodes and lines represent genes and gene interactions, respectively. Numeral number of each network node represents the heating score. Red nodes represent mutated genes while garnet nodes represent propagated genes. Node sizes are proportional to the values of ‘heating scores’ (*i.e.*, the amount of energy gained from network propagation). **D.** Following network propagation, the ‘heating scores’ for the network genes for all the samples are aggregated into a matrix, called netProfile. **E.** The modified MSS algorithm is applied to the netProfile to identify gene signatures that distinguish the two phenotypic groups. Each signature containing a set of genes with heating scores quantifying the network state transition.

Figure 6 shows the flowchart of the eTumorMetastasis. Briefly, somatic mutations were identified using the whole-exome sequencing data of tumors and their paired normal samples. Tools, such as CADD, were applied to the mutations to identify functionally mutated genes (Figure 6A) [18], [19], [20], [21], [22]. Meanwhile, a cancer-specific recurrence network was constructed using gene expression data associated with cancer recurrence and a literature-curated signaling network (Figure 6B). The functionally mutated genes were seeded onto the network to initiate a network propagation that generates ‘heating scores’ for the network genes (Figure 6C). The ‘heating scores’ for the network genes from all the samples were then aggregated into a netMatrix to identify NOG signatures (Figure 6D and E).

To examine whether the sum of somatic mutations in founding clones could predict tumor recurrence, we used breast tumor whole-exome sequencing data. Breast cancer has two major subtypes: ER^+^/luminal and ER^−^/basal, with ER^+^ tumors representing the largest proportion (∼70%) of breast tumors. Through an examination of the sequencing data and clinical follow-up information, we found that TCGA collected several hundreds of ER^+^ tumors but only ∼ 100 ER^−^/basal tumors. We therefore decided to use only ER^+^ tumors in this study. For each sample, we identified functionally mutated genes in the founding clones using the sequencing data of the breast tumors and their paired normal samples (File S1). We then applied eTumorMetastasis (Figure 6; see Method) to identify NOG signatures that are necessary to predict tumor recurrence through a modification of the MSS algorithm [2].

**Figure 7:**
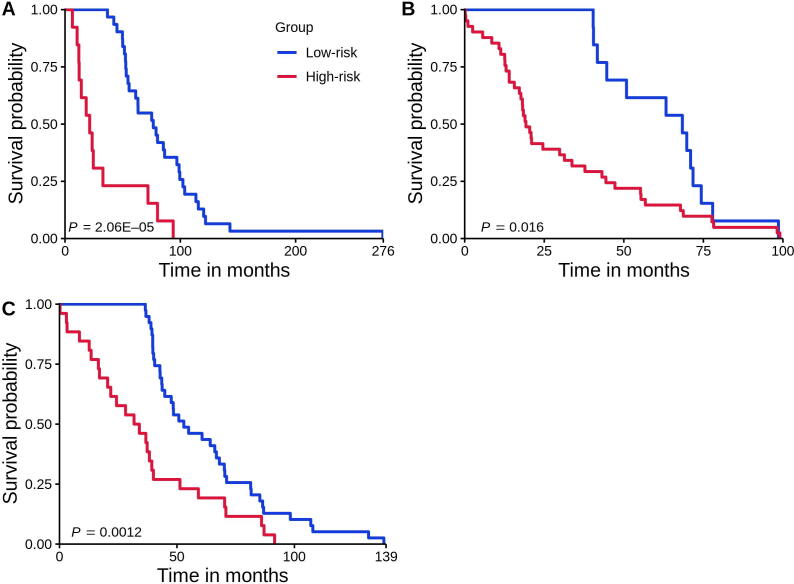
**Kaplan-Meier curves of the risk groups for breast cancer patients with 3-year disease-free survival predicted by the NOG_CSSs A.** NOG_CSSs derived from tumor founding clones’ mutations (*i.e.*, somatic and germline mutations) in the training set. **B.** NOG_CSSs derived from tumor founding clones’ mutations in the validation set 1 (TCGA-Nature). **C.** NOG_CSSs derived from tumor founding clones’ mutations in the validation set 2 (TCGA-CPTAC). Samples without disease-free survival time or who couldn’t be predicted were removed.

We identified 18 NOG signatures and showed that these were significantly predictive in 2 validation sets (Tables S10 and S11). To further improve prediction accuracy, we built NOG_CSSs (Table S6; see Method) using the identified NOG signatures [3]. We showed that founding clone-derived NOG signatures significantly distinguished recurred and non-recurred tumors in the training set of ER^+^ breast cancer patients (Figure 7**A**, *P* = 2.06E−05; Table 1, Table S12). We further showed that the NOG_CSSs (Table 1) significantly distinguished recurred and non-recurred tumors in the validation set 1 (Figure 7B, *P* = 1.6 × 10^−2^; Table 1, Table S12). Finally, we validated these NOG signatures using the independent TCGA-CPTAC set (the validation set 2) which contains 295 additional ER^+^ breast cancer samples (Figure 7C, *P* = 1.2 × 10^−3^; Table 1, Table S12).

**Table 1:** **Prediction accuracy and recall rate for validation sets for breast cancer using the NOG_CSSs derived from tumor founding clones**

Dataset	No. of samples	Low-risk group		High-risk group	
		Precision (%)	Recall (%)	Precision (%)	Recall (%)
Training set	200	90.3	38.2	28.6	13.3
TCGA-Nature (validation set 1)	200	95.2	22.2	13.0	35.0
TCGA-CPTAC (validation set 2)	295	91.9	52.5	21.9	20.6

*Note*: In low-risk group, “Precision” represents the percentage of non-recurred (*i.e.*, non-metastatic) samples in the predicted low-risk group; “Recall” in low-risk group represents the percentage of the predicted low-risk samples from the non-recurred group. In high-risk group, “Precision” represents the percentage of recurred (*i.e.*, metastatic) samples in the predicted high-risk group; “Recall” represents the percentage of the predicted high-risk samples from the recurred group. NOG_CSS, combinatory network operational gene signature set.

To our knowledge, at present, eTumorMetastasis is the only algorithm using genome-wide mutated genes to predict cancer recurrence. Therefore, we decided to compare eTumorMetastasis with Oncotype DX breast cancer test, the only prognostic tool widely used in clinics for ER^+^ breast cancer [13], [16], [17]. Using Oncotype DX, we assigned high-, intermediate-, and low-risk to each sample in our dataset (Table 2, Table S12; File S1; see Method). The results showed that eTumorMetastasis significantly outperformed Oncotype DX for predicting both high- and low-risk groups. For low-risk predictions, the precisions and recalls for eTumorMetastasis were ∼ 5% and ∼ 2%–36% higher than those for Oncotype DX, respectively (Table 1, Table 2). Although high-risk predictions are not important in practice, the precisions of high-risk predictions for eTumorMetastasis were ∼ 6%–10% higher than those for Oncotype DX (Table 1, Table 2). However, because these two models use different input data, we believe the comparison is not optimal, but it is interesting in terms of comparing predictive performance for different data types. Further comparisons will have to be made when new models using genome sequencing emerge.

**Table 2:** **Prediction accuracy and recall rate for validation sets for breast cancer using Oncotype DX model derived from FPKM-UQ RNA-seq data**

Dataset	No. of samples	Low-risk group		High-risk group	
		Precision (%)	Recall (%)	Precision (%)	Recall (%)
Training set	200	84.8	16.6	18.8	40.0
TCGA-Nature (validation set 1)	200	90.0	20.0	6.5	20.0
TCGA-CPTAC (validation set 2)	295	86.0	16.6	10.1	26.5

*Note*: FPKM, fragments per kilobase of transcript per million mapped reads.

## Discussion

Genome sequencing technologies are already being used for personalized genomic tests such as TruGenome clinical sequencing tests (Illumina), personal DNA tests (23andMe), and FoundationOne cancer sequencing tests (Foundation Medicine). We demonstrate that the examination of the collective effects of genomic alterations (*i.e.*, groups of functionally mutated genes) on cancer hallmark networks is more capable of representing the phenotypic consequences of mutated genes. Rare commonalities of mutated genes between tumors suggest that there are multiple ways in which genetic alterations may trigger such a state transition. However, the collective effect of multiple mutated genes may converge into a few sets of network genes (*i.e.*, NOG signatures) that modulate the transition state between tumor recurrence or not. Each of these sets (*i.e.*, NOG signatures) contains a group of genes encoding their regulatory relationships and strengths (represented by heating scores here). In this context, we expect that these NOG signatures might predict tumor recurrence.

Because cancer driver-mutated genes are sparse among tumors, the identification of the NOG signatures requires us to modify this data to estimate their collective impacts on signaling and functional networks. Therefore, we apply a network profiling approach to diffuse the effects of these functionally mutated genes on networks, so that we could identify the subnetworks that are commonly impacted in either the recurred or the non-recurred group (Figure 1). These common network regions provide the means for extracting common features (*i.e.*, genes with similar heating scores) in one group but not in the other group. Of note, in the past, the network propagation algorithm has been mainly used for network topological modeling [23]. Finally, we show that NOG_CSSs derived from the NOG signatures significantly improve the performance of predictive models constructed from genome sequencing data. The concepts here could be widely applicable to other complex diseases for constructing predictive models using genome sequencing data.

At the moment, Oncotype DX is the only predictive model that helps in making clinical decisions for ER^+^ breast cancer in many countries [29]. Here, we show that eTumorMetastasis significantly outperforms Oncotype DX. eTumorMetastasis provides advantages in precision oncology where whole-exome sequencing of tumors not only predicts prognosis (*i.e.*, who will benefit from adjutant chemotherapy) but also optimizes personalized therapy for patients by matching tumor somatic mutated genes. The algorithm also takes into account genome-wide mutated gene profiles to stratify patients’ risk of relapse, whereas many other studies often focus on specific mutations in a single gene. Many key genes such as *BRAF* and *TP53* have been correlated with poor survival outcomes, and yet this provides very limited clinical values when treatment decisions for patients are being explored [30], [31]. However, eTumorMetastasis has been designed for constructing predictive models using mutation data derived from genome or whole-exome sequencing, and thus it is not suitable for other omics data types. In addition, the model also has several limitations. We demonstrate that ER^±^ breast cancer recurrence can be predicted using missense variants. However, for more copy-number driven cancer types (*e.g.*, colon cancer), it is possible that the signal coming from mutations might not be strong enough to predict recurrence accurately. Moreover, there are many other variant types (*e.g.*, stop codon) that affect protein function and could be included in future studies to improve the model.

In summary, it is possible to predict cancer prognosis based on genome sequencing data, which paves the way to the application of genome sequencing technology in clinics. We show that sequencing of a patient’s founding clones might provide an efficient and convenient way for predicting tumor recurrence. Genome sequencing tests could provide cheaper alternatives to current methods used in clinics with similar or even better prognostic values. Finally, the concepts for developing the eTumorMetastasis could be used for predicting clinical outcomes for other complex genetic diseases and cancer types.

## Code availability

eTumorMetastasis pseudocode and related data used in this study can be found at https://github.com/WangEdwinLab/eTumorMetastasis.

## Competing interests

The authors declare no competing interests.

## CRediT authorship contribution statement

**Jean-Sébastien Milanese:** Conceptualization, Methodology, Software, Validation, Formal analysis, Writing – original draft, Writing – review & editing. **Chabane Tibiche:** Conceptualization, Software, Validation. **Naif Zaman:** Conceptualization. **Jinfeng Zou:** Data curation. **Pengyong Han:** Data curation. **Zhigang Meng:** Data curation. **Andre Nantel:** Supervision, Writing – original draft, Writing – review & editing. **Arnaud Droit:** Supervision. **Edwin Wang:** Conceptualization, Methodology, Investigation, Supervision, Writing – original draft, Writing – review & editing.
