# Supplementary material for: eTumorMetastasis: A Network-based Algorithm Predicts Clinical Outcomes Using Whole-exome Sequencing Data of Cancer Patients
Source: Genomics Proteomics Bioinformatics. 2021 Feb 11;19(6):973–85. doi: 10.1016/j.gpb.2020.06.009 (PMC9402585; doi:10.1016/j.gpb.2020.06.009)
Supplement: Supplementary File S1 — Supplementary methods [file mmc1.docx]

**File S1 Supplementary methods**

**Sequencing data pre-processing and variant calling**

The GATK [1] pipeline-based whole exome-sequence data pre-processing was described previously [2]. Briefly, duplicate reads were marked and removed using GATK’s markDuplicates. Reads with a low mapping quality (n = 60) were also removed using Bamtools [3]. Local realignment around indels was made using GATK’s IndelRealign/RealignTargetCreator and finally, base recalibration was conducted using GATK’s BaseRecalibrator. All the variants were obtained using the Varscan2 [4] somatic option by analyzing normal/tumor matched sequencing files. Variants with strand-specific bias, coverage less than 30 reads and variant frequency for heterozygous calls of less than 0.08 were removed.

**Determine tumor purity**

Tumor purity was obtained using absCNseq [5]. To run absCNseq, for a given tumor, we generated a segmentation file and a SNV (Single Nucleotide Variants) file. The segmentation file was generated by running Varscan2 using the standard protocol. Briefly, we ran VarScan2’s copyNumber on normal and tumor BAM files, and VarScan2’s copyCaller to adjust for GC content and finally applied circular binary segmentation. The SNV file was then transformed from the VCF file by running VarScan2. For some samples, absCNseq could give a few purity solutions. In this situation, the consensus purity was selected. The samples with purity greater than 70% were retained for downstream analyses. Ultimately, 755 ER+ breast tumor samples were available for further analysis.

**Training and validation sets for ER+-breast tumors**

To identify gene signatures of ER+ breast cancer, we randomly selected 200 samples, which had follow-up time, as the training set (30 and 170 for recurred and non-recurred samples, respectively). By default, ~15% of ER+ breast tumors get recurred within 10 years which is why we set those group ratios [6]. Currently, in clinics, patient’s classification is often determined by using histology. As a result, patients can be misclassified and, according to their classification, are given a specific treatment route. For example, almost all ER+/luminal breast cancer patients receive tamoxifen treatment. However, tamoxifen treatment for the ‘real’ low-risk patients does not affect patients’ survival and therefore, could be avoided. Specifically to address this issue, in order to develop gene signatures for prognosis, we controlled the training set by selecting ‘low-risk’ patients that were ‘real low-risk’ patients following these rules: (1) low-risk patients who have relatively longer survival in the cohort and (2) we further confirmed their status by predicting them using the gene-expression-based prognostic signatures we developed previously [7]. These signatures were developed using a cohort of patients who did not receive any chemotherapy treatment. We were able to obtain an accuracy of 95% for low-risk ER+ breast cancer prediction. Aside from the training samples, the remaining ER+ breast tumor samples from TCGA were used for validation (Table S2). Sixty samples were retained for obtaining optimal signature cutoffs (Table S6). Finally, all ER+ samples used for validation were separated into 2 sets (TCGA-Nature, TCGA-CPTAC) composed of 200 and 295 samples, respectively. For TCGA-Nature validation set, we used a ratio of 10% of recurred samples (20 recurred and 180 non-recurred samples). For TCGA-CPTAC, we used a ratio of 11.5% of recurred samples (34 recurred and 261 non-recurred samples).

**Comments on eTumorMetastasis algorithm**

*The procedure of modified MSS*

For the recurred and non-recurred samples of the training set, we applied fuzzy clustering to classify the netMatrix into 2 classes and then conducted a log-rank test to identify modulated genes (*P* < 0.05) based on genes’ heating scores. From these modulated genes, we collected hallmark GO-defined genes. For each cancer-hallmark GO-defined, we collected between 100 and 200 genes. From the training set, we generated 200 random netMatrix sets and generated 5 million random gene sets (30 genes per set). Then, we ran a fuzzy clustering analysis (k = 2) for each random netMatrix sets (200) using each gene set (5 million) to distinguish low- and high-risk groups (log-rank test, *P* < 0.05). For a given cancer hallmark GO term, if 1000−5000 random gene sets were able to distinguish low- and high-risk groups (log-rank test, *P* < 0.05) in more than 90% of the 200 random netMatrix sets (*P* < 0.05), we collected and ranked the genes based on their frequency among the significant gene-sets. The top ranked genes (30 genes) were used as a NOG gene signature for that cancer hallmark.

*Determine which hallmark-based GO term could generate a NOG gene signature*

For a given cancer hallmark-based GO term, the number of the significant gene sets is greater than 1000 but less than 5000 (from 5 million gene sets, *P* < 0.001). If the number of gene sets was lower than 1000, the signature obtained from the gene frequency was not robust enough. If the number of gene sets was greater than 5000, we set a more stringent *P* value cutoff.

*Robustness of the NOG gene signatures*

There is 3 major components of the eTumorMetastasis that make its gene signatures more robust than any other methods: (1) Higher genome instability in either germline or tumor genomes creates more mutations. Although some of these mutations are functionally mutated, most of them have no direct impact on cancer biology and act as background noise. These mutations will generate many 'passenger signals' in the netMatrix when we applied the network propagation approach. Thus, the variability of the netMatrix between individual founding clones can be very high, and the cancer-related signals (*e.g.*, heating scores) may be buried in those highly mutational profiles. To cope with this problem, we only focused on cancer-hallmark associated genes which are most likely to be associated with cancer recurrence. Therefore, the NOG gene signatures derived from cancer-hallmark associated genes are most likely signals related to cancer and will hold more robustness when testing on independent data sets; (2) The significant gene-sets, which are able to distinguish recurred and non-recurred samples among 90% of the 200 random datasets, were generated from the original training set. Therefore, the selected significant gene sets have higher robustness; (3) Finally, by collecting between 1000-5000 significant gene-sets from a pool of 5 million random gene sets (*P* < 0.001) and by ranking genes in those sets, we provide much more robustness for the NOG gene signatures.

**Oncotype DX formula calculation**

The recurrence score (RS) for all samples for tumoral RNA-seq was calculated based on previous publications [8−10]. Proteins were transformed into genes:

HER2 Group Score (0.9 x *GRB7*) + (0.1 x *ERBB2*)

If HER2 Group Score is less than 8 then the HER2 Group Score is considered equal to 8

# ER Group Score ([0.8 x *ESR1*] + [1.2 x *PGR*] + *BCL2* + *SCUBE2*)/4

Proliferation Group Score (*BIRC5* + *MKI67* + *MYBL2* + *CCNB1* + *AURKA*)/5

If the Proliferation Group Score is under 6.5 then the Proliferation Group Score is considered equal to 6.5

Invation Group Score (*CTSV* + *MMP11*)/2

RS_U_ = + 0.47 x HER2 Group Thresholded Score

- 0.34 x ER Group Score

+ 1.04 x Proliferation Group Thresholded Score

+ 0.10 x Invasion Group Score

+ 0.05 x *CD68*

- 0.08 x *GSTM1*

- 0.07 x *BAG1*

Cutoffs for groups were initially set as RS < 18 for low-risk, RS ≥ 37 for high-risk and RS between 18 and 37 for intermediate-risk with a RS scale between 0 and 100. Because our RS scale was different than the ones obtained for RT-PCR, we had to adjust these cutoffs accordingly. Samples were sorted based on their RS. Bottom 17% of samples were assigned to the low-risk group (lowest RS), top 37% of samples were assigned to the high-risk group (highest RS) and finally, all other samples were assigned to the intermediate-risk group.

**Statistical analysis**

Statistical significance of the prognostic groups (*i.e.*, high- or low-risk groups defined by gene signatures) was determined using Kaplan-Meier survival plots. A prognostically significant result was defined by log-rank *P* < 0.05. Prognostic significance of clinicopathologic factors and molecular features (*i.e.*, mutated genes) were performed with the use of the Cox proportional hazards regression model. P-values were based on likelihood ratio tests. All the analyses were performed using the statistical R package. Survival curves were created using the survival and survminer R package.

**References**

[1] DePristo MA, Banks E, Poplin R, Garimella KV, Maguire JR, Hartl C, et al. A framework for variation discovery and genotyping using next-generation DNA sequencing data. Nat Genet 2011;43:491−8.

[2] Zaman N, Li L, Jaramillo ML, Sun Z, Tibiche C, Banville M, et al. Signaling network assessment of mutations and copy number variations predict breast cancer subtype-specific drug targets. Cell Rep 2013;5:216−23.

[3] Barnett DW, Garrison EK, Quinlan AR, Strömberg MP, Marth GT. BamTools: a C++ API and toolkit for analyzing and managing BAM files. Bioinformatics 2011;27:1691−2.

[4] Koboldt DC, Zhang Q, Larson DE, Shen D, McLellan MD, Lin L, et al. VarScan 2: somatic mutation and copy number alteration discovery in cancer by exome sequencing. Genome Res 2012;22:568−76.

[5] Bao L, Pu M, Messer K. AbsCN-seq: a statistical method to estimate tumor purity, ploidy and absolute copy numbers from next-generation sequencing data. Bioinformatics 2014;30:1056−63.

[6] Voduc KD, Cheang MC, Tyldesley S, Gelmon K, Nielsen TO, Kennecke H. Breast cancer subtypes and the risk of local and regional relapse. J Clin Oncol 2010;28:1684−91.

[7] Li J, Lenferink AE, Deng Y, Collins C, Cui Q, Purisima EO, et al*.* Identification of high-quality cancer prognostic markers and metastasis network modules. Nat Commun 2010;1:34.

[8] Paik S, Tang G, Shak S, Kim C, Baker J, Kim W, Cronin M, et al. A multigene assay to predict recurrence of tamoxifen-treated, node-negative breast cancer. N Engl J Med 2004;351:2817−26.

[9] Paik S, Tang G, Shak S, Kim C, Baker J, Kim W, et al. Gene expression and benefit of chemotherapy in women with node-negative, estrogen receptor-positive breast cancer. J Clin Oncol 2006;24:3726−34.

[10] Sinicropi D, Qu K, Collin F, Crager M, Liu ML, Pelham RJ, et al. Whole transcriptome RNA-seq analysis of breast cancer recurrence risk using formalin-fixed paraffin-embedded tumor tissue. PLoS One 2012;7:e40092.
