# Supplementary material for: eTumorMetastasis: A Network-based Algorithm Predicts Clinical Outcomes Using Whole-exome Sequencing Data of Cancer Patients
Source: Genomics Proteomics Bioinformatics. 2021 Feb 11;19(6):973–85. doi: 10.1016/j.gpb.2020.06.009 (PMC9402585; doi:10.1016/j.gpb.2020.06.009)
Supplement: Supplementary Table 1 [file mmc2.docx]

**Table S1 Demographic and clinical characteristics for ER+ breast cancer samples**

| **Clinical characteristic** | **Training set (n = 200)** | |  | **Validation set 1**  **TCGA-CPTAC**  **(n = 295)** | |  | **Validation set 2**  **TCGA nature**  **(n = 200)** | |
| --- | --- | --- | --- | --- | --- | --- | --- | --- |
|  | **Number of patients** | **%** |  | **Number of patients** | **%** |  | **Number of patients** | **%** |
| Age, years |  |  |  |  |  |  |  |  |
| Median | 59 | |  | 60 | |  | 58 | |
| ≤ median | 102 | 51 |  | 149 | 50.51 |  | 105 | 52.5 |
| > median | 98 | 49 |  | 146 | 49.49 |  | 95 | 47.5 |
| Death |  |  |  |  |  |  |  |  |
| Yes | 29 | 14.5 |  | 33 | 11.19 |  | 26 | 13 |
| No | 171 | 85.5 |  | 262 | 88.81 |  | 174 | 87 |
| Localization |  |  |  |  |  |  |  |  |
| Left | 102 | 51 |  | 146 | 49.49 |  | 113 | 56.5 |
| Right | 98 | 49 |  | 149 | 50.51 |  | 87 | 43.5 |
| Stage |  |  |  |  |  |  |  |  |
| I | 37 | 18.5 |  | 53 | 17.97 |  | 30 | 15 |
| II | 108 | 54 |  | 164 | 55.59 |  | 113 | 56.5 |
| III | 40 | 20 |  | 72 | 24.4 |  | 49 | 24.5 |
| IV | 8 | 4 |  | 2 | 0.68 |  | 4 | 2 |
| X | 5 | 2.5 |  | 2 | 0.68 |  | 3 | 1.5 |
| NA | 2 | 1 |  | 2 | 0.68 |  | 1 | 0.5 |
| Subtype |  |  |  |  |  |  |  |  |
| Luminal A | 95 | 47.5 |  | 38 | 12.88 |  | 58 | 29 |
| Luminal B | 42 | 21 |  | 18 | 6.1 |  | 46 | 23 |
| Unknown | 10 | 5 |  | 11 | 3.73 |  | 21 | 10.5 |
| NA | 53 | 26.5 |  | 228 | 77.29 |  | 75 | 37.5 |
| Relapse |  |  |  |  |  |  |  |  |
| Yes | 30 | 15 |  | 34 | 11.5 |  | 20 | 10 |
| No | 170 | 85 |  | 261 | 88.5 |  | 180 | 90 |

*Note*: NA, not available.
