# Supplementary material for: eTumorMetastasis: A Network-based Algorithm Predicts Clinical Outcomes Using Whole-exome Sequencing Data of Cancer Patients
Source: Genomics Proteomics Bioinformatics. 2021 Feb 11;19(6):973–85. doi: 10.1016/j.gpb.2020.06.009 (PMC9402585; doi:10.1016/j.gpb.2020.06.009)
Supplement: Supplementary Table 2 [file mmc3.docx]

**Table S2 Sample filtering steps in breast cancer dataset**

| **Dataset** | **Clinical information** | **Sequencing** | **Training set** | **Cutoff set** | **Validation set 1*** | **Validation set 2*** |
| --- | --- | --- | --- | --- | --- | --- |
| Breast | 1067 | 755 | 200 | 60 | 295 | 200 |

*Note*: *, validation set 1 and 2 refers to the TCGA-CPTAC and TCGA nature sets, respectively.
