# Supplementary material for: eTumorMetastasis: A Network-based Algorithm Predicts Clinical Outcomes Using Whole-exome Sequencing Data of Cancer Patients
Source: Genomics Proteomics Bioinformatics. 2021 Feb 11;19(6):973–85. doi: 10.1016/j.gpb.2020.06.009 (PMC9402585; doi:10.1016/j.gpb.2020.06.009)
Supplement: Supplementary Table 6 [file mmc7.docx]

**Table S6 Constructing founding clone-based NOG_CSSs for ER+ breast cancer by combining the training set and the cutoff set**

| **Number of**  **Signatures** | **Low-risk** | |  | **High-risk** | |
| --- | --- | --- | --- | --- | --- |
|  | **Accuracy (%)^*^** | **Recall (%)^†^** |  | **Accuracy (%)^**^** | **Recall (%)^††^** |
| 1 | 85.00 | 100.00 |  | 15.12 | 100.00 |
| 2 | 84.77 | 98.19 |  | 15.18 | 100.00 |
| 3 | 85.20 | 96.38 |  | 15.08 | 97.44 |
| 4 | 86.19 | 93.21 |  | 15.35 | 94.87 |
| 5 | 86.40 | 89.14 |  | 15.79 | 92.31 |
| 6 | 87.98 | 82.81 |  | 16.20 | 89.74 |
| 7 | 89.95 | 76.92 |  | 17.86 | 89.74 |
| 8 | 91.67 | 69.68 |  | 18.39 | 82.05 |
| 9 | 91.43 | 57.92 |  | 19.33 | 74.36 |
| 10 | 90.91 | 45.25 |  | 22.50 | 69.23 |
| 11 | 91.86 | 35.75 |  | 27.17 | 64.10 |
| 12 | 93.75 | 27.15 |  | 28.17 | 51.28 |
| 13 | 90.91 | 18.10 |  | 26.92 | 35.90 |
| 14 | 90.63 | 13.12 |  | 25.00 | 20.51 |
| 15 | 89.47 | 7.69 |  | 28.57 | 15.38 |
| 16 | 87.50 | 3.17 |  | 20.00 | 5.13 |
| 17 | 100.00 | 1.36 |  | 0.00 | 0.00 |
| 18 | 100.00 | 0.90 |  | 0.00 | 0.00 |

*Note*: *, percentage of non-recurred (*i.e.*, non-metastatic) samples in the predicted low-risk group. †, percentage of the predicted low-risk samples from the non-recurred group. **, percentage of recur (*i.e.*, metastatic) samples in the predicted high-risk group. ††, percentage of the predicted high-risk samples from the recurred group. Cutoffs selected are highlighted.
