# Supplementary material for: eTumorMetastasis: A Network-based Algorithm Predicts Clinical Outcomes Using Whole-exome Sequencing Data of Cancer Patients
Source: Genomics Proteomics Bioinformatics. 2021 Feb 11;19(6):973–85. doi: 10.1016/j.gpb.2020.06.009 (PMC9402585; doi:10.1016/j.gpb.2020.06.009)
Supplement: Supplementary Table 9 [file mmc10.docx]

**Table S9 Prediction accuracy and recall rate for validation sets for breast cancer using Oncotype DX model derived from FPKM RNA-seq data**

| **Dataset** | **Number of samples** | **Low-risk** | |  | **High-risk** | |
| --- | --- | --- | --- | --- | --- | --- |
|  |  | **Precision (%)*** | **Recall (%)^†^** |  | **Precision (%)**** | **Recall (%)^††^** |
| Training set | 200 | 87.5 | 16.7 |  | 18.2 | 40.0 |
| TCGA-Nature | 200 | 90.0 | 20.0 |  | 5.0 | 15.0 |
| TCGA-CPTAC | 295 | 84.0 | 16.2 |  | 11.2 | 29.4 |

*Note*: *, percentage of non-recurred (*i.e.*, non-metastatic) samples in the predicted low-risk group. †, percentage of the predicted low-risk samples from the non-recurred group. **, percentage of recurred (*i.e.*, metastatic) samples in the predicted high-risk group. ††, percentage of the predicted high-risk samples from the recurred group.
