# Supplementary material for: eTumorMetastasis: A Network-based Algorithm Predicts Clinical Outcomes Using Whole-exome Sequencing Data of Cancer Patients
Source: Genomics Proteomics Bioinformatics. 2021 Feb 11;19(6):973–85. doi: 10.1016/j.gpb.2020.06.009 (PMC9402585; doi:10.1016/j.gpb.2020.06.009)
Supplement: Supplementary Table 11 [file mmc12.docx]

**Table S11 Prediction accuracy and recall rate for the network operational gene signatures derived from founding clones’ mutations of breast cancer tumors**

| **Dataset** | **Number of samples** | **Cancer Hallmark** | **Low-risk** | |  | **High-risk** | |
| --- | --- | --- | --- | --- | --- | --- | --- |
|  |  |  | **Accuracy (%)*** | **Recall (%)^†^** |  | **Accuracy (%)**** | **Recall (%)^††^** |
| Training | 200 | Apoptosis 1 | 81.7 | 57.7 |  | 10.0 | 26.7 |
|  |  | Apoptosis 2 | 98.4 | 35.9 |  | 21.0 | 96.7 |
|  |  | Apoptosis 3 | 98.9 | 51.8 |  | 26.1 | 96.7 |
|  |  | Cell Cycle 1 | 88.6 | 54.7 |  | 19.0 | 60.0 |
|  |  | Cell Cycle 2 | 91.0 | 53.5 |  | 21.0 | 70.0 |
|  |  | Cell Cycle 3 | 95.7 | 51.8 |  | 24.1 | 86.7 |
|  |  | Cell Adhesion 1 | 87.5 | 57.7 |  | 18.2 | 53.3 |
|  |  | Cell Adhesion 2 | 91.5 | 57.1 |  | 22.3 | 70.0 |
|  |  | Cell Adhesion 3 | 93.2 | 56.7 |  | 23.7 | 76.7 |
|  |  | Cytoskeleton 1 | 94.1 | 56.5 |  | 24.5 | 80.0 |
|  |  | Cytoskeleton 2 | 93.3 | 49.4 |  | 21.8 | 80.0 |
|  |  | Cytoskeleton 3 | 100.0 | 42.4 |  | 23.4 | 100.0 |
|  |  | Immune Response 1 | 80.2 | 45.3 |  | 10.6 | 36.7 |
|  |  | Immune Response 2 | 83.9 | 45.9 |  | 14.0 | 50.0 |
|  |  | Immune Response 3 | 89.6 | 55.9 |  | 20.2 | 63.3 |
|  |  | Cell Proliferation 1 | 87.7 | 54.7 |  | 18.1 | 56.7 |
|  |  | Cell Proliferation 2 | 84.5 | 51.2 |  | 14.4 | 46.7 |
|  |  | Cell Proliferation 3 | 91.2 | 60.6 |  | 23.0 | 66.7 |
| TCGA-Nature | 200 | Apoptosis 1 | 98.0 | 27.2 |  | 12.7 | 95.0 |
|  |  | Apoptosis 2 | 90.9 | 38.9 |  | 10.6 | 65.0 |
|  |  | Apoptosis 3 | 91.9 | 37.8 |  | 11.1 | 70.0 |
|  |  | Cell Cycle 1 | 85.3 | 28.9 |  | 7.9 | 55.0 |
|  |  | Cell Cycle 2 | 88.9 | 35.6 |  | 9.4 | 60.0 |
|  |  | Cell Cycle 3 | 92.9 | 36.1 |  | 11.5 | 75.0 |
|  |  | Cell Adhesion 1 | 91.6 | 42.2 |  | 11.1 | 65.0 |
|  |  | Cell Adhesion 2 | 94.1 | 44.4 |  | 13.0 | 75.0 |
|  |  | Cell Adhesion 3 | 91.0 | 45.0 |  | 10.8 | 60.0 |
|  |  | Cytoskeleton 1 | 90.9 | 22.2 |  | 10.3 | 80.0 |
|  |  | Cytoskeleton 2 | 89.9 | 39.4 |  | 9.9 | 60.0 |
|  |  | Cytoskeleton 3 | 88.7 | 47.8 |  | 8.7 | 45.0 |
|  |  | Immune Response 1 | 91.8 | 43.3 |  | 11.3 | 65.0 |
|  |  | Immune Response 2 | 92.2 | 39.4 |  | 11.4 | 70.0 |
|  |  | Immune Response 3 | 91.3 | 40.6 |  | 10.8 | 65.0 |
|  |  | Cell Proliferation 1 | 87.3 | 38.3 |  | 8.3 | 50.0 |
|  |  | Cell Proliferation 2 | 90.6 | 42.8 |  | 10.4 | 60.0 |
|  |  | Cell Proliferation 3 | 86.6 | 39.4 |  | 7.6 | 45.0 |
| TCGA-CPTAC | 295 | Apoptosis 1 | 93.1 | 67.1 |  | 19.6 | 61.8 |
|  |  | Apoptosis 2 | 91.3 | 44.1 |  | 13.6 | 67.7 |
|  |  | Apoptosis 3 | 93.6 | 55.6 |  | 17.1 | 70.6 |
|  |  | Cell Cycle 1 | 89.1 | 62.5 |  | 12.5 | 41.2 |
|  |  | Cell Cycle 2 | 89.9 | 58.2 |  | 13.5 | 50.0 |
|  |  | Cell Cycle 3 | 91.6 | 62.5 |  | 16.2 | 55.9 |
|  |  | Cell Adhesion 1 | 90.3 | 60.5 |  | 14.2 | 50.0 |
|  |  | Cell Adhesion 2 | 90.1 | 55.6 |  | 13.4 | 52.9 |
|  |  | Cell Adhesion 3 | 90.5 | 54.8 |  | 13.9 | 55.9 |
|  |  | Cytoskeleton 1 | 94.4 | 71.3 |  | 23.5 | 67.7 |
|  |  | Cytoskeleton 2 | 86.5 | 46.7 |  | 9.7 | 44.1 |
|  |  | Cytoskeleton 3 | 88.1 | 45.2 |  | 11.2 | 52.9 |
|  |  | Immune Response 1 | 91.7 | 59.0 |  | 15.8 | 58.8 |
|  |  | Immune Response 2 | 92.7 | 62.8 |  | 17.8 | 61.8 |
|  |  | Immune Response 3 | 91.7 | 63.2 |  | 16.5 | 55.9 |
|  |  | Cell Proliferation 1 | 85.3 | 46.7 |  | 8.6 | 38.2 |
|  |  | Cell Proliferation 2 | 89.1 | 50.2 |  | 12.2 | 52.9 |
|  |  | Cell Proliferation 3 | 88.4 | 46.7 |  | 11.5 | 52.9 |

*Notes*: *, percentage of non-recurred (i.e., non-metastatic) samples in the predicted low-risk group. †, percentage of the predicted low-risk samples from the non-recurred group. **, percentage of recurred (i.e., metastatic) samples in the predicted high-risk group. ††, percentage of the predicted high-risk samples from the recurred group.
