# Supplementary material for: eTumorMetastasis: A Network-based Algorithm Predicts Clinical Outcomes Using Whole-exome Sequencing Data of Cancer Patients
Source: Genomics Proteomics Bioinformatics. 2021 Feb 11;19(6):973–85. doi: 10.1016/j.gpb.2020.06.009 (PMC9402585; doi:10.1016/j.gpb.2020.06.009)
Supplement: Supplementary Table 12 [file mmc13.docx]

**Table S12 Prediction accuracy and recall rate for validation sets for breast cancer using the NOG_CSSs derived from tumor founding clones**

| **Dataset** | **Number of samples** | **Low-risk** | |  | **Intermediate-risk** | |  |  | **High-risk** | |
| --- | --- | --- | --- | --- | --- | --- | --- | --- | --- | --- |
|  |  | **Precision (%)*** | **Recall (%)^†^** |  | **Precision (%)**** | **Recall (%)^††^** |  |  | **Precision (%)***** | **Recall (%)^†††^** |
| Training set | 200 | 90.3 | 38.2 |  | 83.3 | 55.9 |  |  | 28.6 | 13.3 |
| TCGA-Nature | 200 | 95.2 | 22.2 |  | 89.4 | 51.7 |  |  | 13.0 | 35.0 |
| TCGA-CPTAC | 295 | 91.9 | 52.5 |  | 86.8 | 37.9 |  |  | 21.9 | 20.6 |

*Note*: *, percentage of non-recurred (*i.e.*, non-metastatic) samples in the predicted low-risk group. †, percentage of the predicted low-risk samples from the non-recurred group. **, percentage of non-recurred (*i.e.*, non-metastatic) samples in the predicted intermediate-risk group. ††, percentage of the predicted intermediate-risk samples from the non-recurred group. ***, percentage of recurred (*i.e.*, metastatic) samples in the predicted high-risk group. †††, percentage of the predicted high-risk samples from the recurred group.
